# Length of hospital stay and associated factors among adult surgical patients admitted to surgical wards in Amhara Regional State Comprehensive Specialized Hospitals, Ethiopia

**DOI:** 10.1371/journal.pone.0296143

**Published:** 2024-08-12

**Authors:** Habtamu Hurisa Dadi, Netsanet Habte, Yenework Mulu

**Affiliations:** 1 Department of Surgical Nursing, School of Nursing, College of Health Sciences and Medicine, Wolaita Sodo University, Wolaita Sodo, Ethiopia; 2 Department of Adult Health Nursing, School of Nursing, College of Medicine and Health Sciences, University of Gondar, Gondar, Ethiopia; 3 Department of Surgical Nursing, School of Nursing, College of Medicine and Health Sciences, University of Gondar, Gondar, Ethiopia; Monash University, ETHIOPIA

## Abstract

**Introduction:**

Hospitals across the country are facing increases in hospital length of stay ranging from 2% to 14%. This results in patients who stay in hospital for long periods of time being three times more likely to die in hospital. Therefore, identifying factors that contribute to longer hospital stays enhances the ability to improve services and quality of patient care. However, there is limited documented evidence on factors associated with longer hospital stays among surgical inpatients in Ethiopia and the study area.

**Objective:**

This study aimed to assess the length of hospital stay and associated factors among **adult surgical** patients admitted to surgical wards in Amhara Regional State Comprehensive Specialized Hospitals, Ethiopia, 2023.

**Methods:**

An institutional-based cross-sectional study was conducted among 452 adult surgical patients from April 17 to May 22, 2023. Data were collected based on a pretested, structured, interviewer-administered questionnaire, medical record review, and direct measurement of BMI. Study participants were selected using a systematic random sampling technique. The collected data were cleaned, entered into EpiData version 4.6.0 and exported to STATA version 14 for analysis. Binary logistic regression analysis was used. Variables with a p value <0.05 in the multivariable logistic regression analysis were considered statistically significant.

**Results:**

In the current study, the prevalence of prolonged hospital stay was 26.5% (95% CI: 22.7, 30.8). Patients referred from another public health facility (AOR = 2.65; 95% CI: 1.14, 6.14), hospital-acquired pneumonia (AOR = 3.64; 95% CI: 1.43, 9.23), duration of surgery ≥110 minutes (AOR = 2.54; 95% CI: 1.25, 5.16), being underweight (AOR = 5.21; 95%CI: 2.63, 10.33) and preoperative anemia (AOR = 3.22; 95% CI: 1.77, 5.86) were factors associated with prolonged hospital stays.

**Conclusion:**

This study found a significant proportion of prolonged hospital stays among patients admitted to surgical wards. Patients referred from another public health facility, preoperative anemia, underweight, duration of surgery ≥110 minutes, and hospital-acquired pneumonia were factors associated with prolonged hospital stay. Early screening and treatment of anemia and malnutrition before surgery can shorten the length of stay.

## Introduction

Hospitalization is critical for improving patient care and providing inpatient services, which can help save lives [1]. Hospitals significantly affect the efficiency of healthcare systems [2]. Length of stay (LoS) is a marker of hospital indicators that is currently utilized regularly to evaluate hospital efficiency and is defined as the date between admission and discharge [3–5]. In contrast, prolonged LoS is defined as a hospital stay longer than expected for particular treatments [6]. PLoS definitions vary across the literature owing to the use of different cutoff points, such as above the median [7], 75^th^ [1, 8, 9], or 95^th^ percentiles [10]. Although the term has not been standardized, these patients have poorer outcomes, both from a health and socioeconomic perspective [10].

Every year, more than 143 million people in low- and middle-income countries undergo surgical procedures, which places substantial strain on the healthcare system [11]. As a result, hospitals across the country are facing an increase in length of hospital stay. Only 2% of patients in 2012 had hospital stays longer than 21 days; currently, they account for 14% of hospital days and cost more than $20 billion a year [12]. In 2022, patients’ median hospitalizations would be nearly 19% higher than they were in 2019, according to statistics from the healthcare consulting company Strata Decision Technology [13]. In European hospitals, 7.98% to 16.67% of patients experienced a prolonged length of hospital stay [14–16]. In Asia, studies have shown that 5.4% to 30.5% of patients had prolonged hospital stays [9, 17, 18], and in Africa, 24.7% to 63% of patients experienced prolonged hospital stays [8, 19, 20].

Hence, hospitals face significant challenges related to insufficient surgical bed capacity and inconsistent occupancy rates in surgical units, resulting in frequent last-minute cancellations of surgeries [21]. This has a significant impact on patients, their families, and health services [1, 7, 16]. Moreover, patients who stayed for a prolonged time had poor care outcomes [22, 23], faced financial burdens [24, 25], made it difficult for the hospital to run at a high level of efficiency [26, 27], were at a higher risk of developing complications [28, 29], and had an increased mortality rate [14].

Studies have revealed that factors such as older age, preoperative hypoalbuminemia, ASA class 3 or 4, duration of operation, in-hospital complications, comorbidity, and reoperation were independently associated with prolonged hospital stays [8, 22, 25, 30, 31]. However, there is a lack of consistency in what influences patients’ LoS, particularly in surgical wards [32]. On the other hand, this research topic covers a number of previously unexplored aspects that have attracted research interest, such as postponed surgery, preoperative anemia, functional status, and source of referral [8, 19], which should be investigated further. An investigation of these factors is necessary to reduce the length of hospital stay.

In an effort to enhance patient care quality and lower hospital costs, many parts of the health system are working hard to reduce unnecessary hospital stays [33, 34] through interdisciplinary or multidisciplinary care, medication management, and discharge planning tools [32, 34]. As in other countries, the Ethiopian Federal Ministry of Health (EFMoH) is trying to improve the quality of surgical care by launching a strategy to save lives through safe surgery, and has implemented the World Health Organization’s surgical safety checklist, which has an effect on reducing the length of hospital stay [35, 36].

Although the length of stay (LoS) of patients admitted to surgical units has been declining in Western nations, it remains high in Africa [8, 19, 20]. This disparity is a result of the varied healthcare systems and clinical practices [37] and may also be due to the lack of utilization of strategies implemented in hospitals related to surgical care, which influences the length of hospital stay. Moreover, there are inconsistent findings regarding the efficacy of LoS-reduction measures, such as discharge planning tools, which are usually utilized by healthcare systems [34]. In addition, there is little published literature on the factors that influence the length of hospital stays of among surgical inpatients in Africa and Ethiopia. Therefore, this study aimed to assess the length of hospital stay and associated factors among adult surgical patients admitted to surgical wards in the Amhara Regional State comprehensive specialized hospitals in Ethiopia. The results of these findings provide additional clues for the Ethiopian healthcare system to gain a better understanding of the factors contributing to prolonged hospital stays with surgical care services.

## Materials and methods

### Study design, period and setting

An institutional cross-sectional study was conducted on patients admitted to the adult surgical wards in Amhara Regional State Comprehensive Specialized Hospitals from April 17 to May 22, 2023. There are eight comprehensive specialized hospitals in Amhara Regional States. University of Gondar, Dessie, Felege Hiwot and Tibebe Ghion were the four selected comprehensive specialized hospitals. According to reports from the Human Resources Department and Health Management Information System (HMIS), these hospitals currently have more than 2,900 healthcare professionals, including more than 126 nurses, working in the surgical wards and have 277 beds in the surgical wards.

The University of Gondar is a comprehensive specialized hospital in the Amhara regional state. This hospital serves more than 5 million people in its catchment area. In the hospital, there were more than 906 healthcare professionals (463 nurses) and had 40 beds in surgical ward. The average monthly admission rate of surgical patients was 106.

The Tibebe Ghion and Felege Hiwot comprehensive specialized hospitals are located in Bahir Dar, the capital of Amhara regional state, which is 565 km from Addis Ababa and serves more than five million people in each hospital [38]. In the surgical ward, they had 105 and 66 beds, respectively. The average monthly number of surgical patient admissions was 335 and 168, respectively.

Dessie Comprehensive Specialized Hospital in Northeast Ethiopia. The municipality of Dessie is located far from Bahir Dar (481 km), the capital of Amhara regional state, and 401 km from Addis Ababa, the capital of Ethiopia. It serves a population of 5 million people, employs 603 healthcare workers and has 66 surgical ward beds. The average monthly admission of surgical patients was 141.

### Population

All adult (≥18 years) surgical patients who admitted to surgical wards in the Amhara Regional State CSHs were the source population, and all adult (≥18 years) surgical patients who were admitted to surgical wards in selected Amhara Regional State CSHs during the study period were the study population.

## Inclusion and exclusion criteria

All sampled adult surgical patients who underwent both elective and emergency surgery and were admitted to surgical wards in selected CSHs of Amhara Regional State were included. The study excluded patients who were unable to communicate because of their illness without available caregivers and orthopedic patients.

### Sample size determination

#### Sample size determination for proportion

The sample size was determined using a single population proportion formula with the following assumptions:

n = sample size

Z α/2 = significance level = 95% CI = 1.96, d = margin of error of 0.05, and proportion of prolonged length of hospital stay from a previous study at Jimma Hospital (P), 25.3% [19].

n = (Z α/2) ^2^ p (1-p) = (1.96)^2^ 0.253 (0.747) = 290

d^2^ (0.05)^2^

By considering 1.5 design effects and adding a 5% (22) nonresponse rate, the total sample size was 457.

#### Sample size determinations for factors

The sample sizes for the second objective were calculated using EpiInfo version 7.2 with the following assumptions: 95% confidence interval, 80% power of the study, 1:1 ratio of exposed to non-exposed groups, adjusted odds ratio, and percentage of outcomes in exposed and unexposed groups from previous studies, as shown in the table below. As the study design was cross-sectional, an odds ratio was used rather than another measure of association “Table 1”.

**Table 1:** Sample size determination for factors associated with length of hospital stay.

Factors	Assumption	Proportion	Initial sample sizes	1.5 Design effects with 5% NRR	References
Surgical site infection	CI = 95% Power = 80% Ratio 1:1	P1 = 5.4% P2 = 19.3%	68	107	[8]
Hospital acquired pneumonia		P1 = 4.1% P2 = 71.2%	168	265	
Comorbidity		P1 = 12.3% P2 = 12.3%	96	151	
Health insurance		P1 = 34.8% P2 = 23.3%	130	205	[19]

NRR, Non-response rate; CI, confidence interval

Based on the results, the proportion of patients with a prolonged length of hospital stay was used for the final sample size.

### Sampling procedure

**Fig 1:**
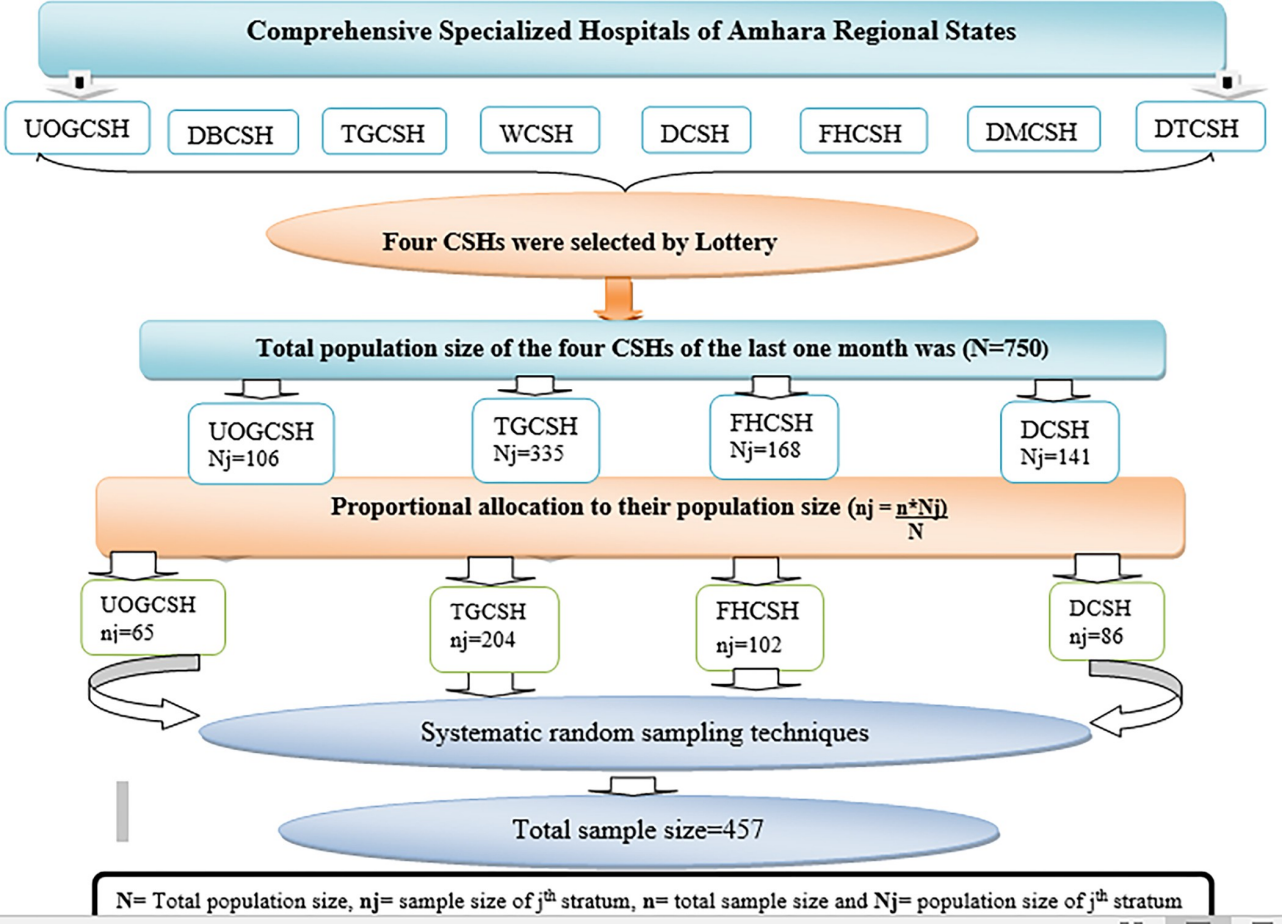
Schematic presentation of sampling procedures for patients admitted to surgical wards in Amhara Regional States Comprehensive Specialized Hospitals, Ethiopia, 2023.

Out of eight comprehensive specialized hospitals in the Amhara regional states, four were selected by lottery, including the University of Gondar, Dessie, Felege Hiwot and Tibebe Ghion comprehensive specialized hospitals. The total number of patients who underwent surgery and were admitted to the surgical wards of these hospitals in the month before data collection was 750. Then, the sample size was proportionally distributed based on their population size using the proportional allocation formula. Study participants were then recruited using systematic random selection. The first study participant was then selected for the survey by lottery and every other study participants were selected until the required sample size was reached “Fig 1”.

### Study variables

#### Dependent variable

Length of hospital stays (Prolonged, Normal)

#### Independent variables

Sociodemographic factors, admission related, clinical characteristic and behavioral factors

### Operational definitions

**Length of hospital stay:** described as the length of a single hospitalization, which is determined by subtracting the day of admission from the day of discharge.

**Prolonged LoS:** defined as an LoS greater than or equal to the 75^th^ percentile in hospitals for the entire study population that was at least 14 days in this study [19].

**Normal LoS:** defined as LoS less than the 75^th^ percentile for the entire study population

**In-hospital complications:** The occurrence of new medical issues upon admission that were seen as negative incidents arising from the course of care and therapy rather than the course of the disease, such as pressure injuries, falls, healthcare-associated infections (HAIs), surgical complications, respiratory complications, cardiac complications, venous thromboembolism, renal failure, gastrointestinal bleeding, and adverse drug events. HAIs were further classified into specific types, such as central-line associated bloodstream, catheter-associated urinary tract infection, hospital-acquired pneumonia, ventilator-associated pneumonia, and surgical site infections [39].

**Preoperative anemia:** defined as hemoglobin (Hb) levels below 12.0 g/dL in women and 13.0 g/dL in males, was considered anemia, according to the World Health Organization (WHO) [40].

**Smoking status:** Patients were classified as never smokers if they had never smoked, current smokers if they smoked at least one cigarette per day prior to hospitalization, and former smokers if they had smoked frequently or intermittently in the past [41].

**Alcohol drinking status:** classified as never for those who never consumed any alcoholic drinks; current drinkers were all those that consumed alcohols prior to hospitalization; past alcohol drinkers were those persons who consumed any alcoholic drink in the past but stopped any consumption in the past year [42].

**The duration of surgery:** was defined as the time from the first incision to wound closure [43].

**Wealth index:** determined by using the Principal Component Analysis (PCA) by considering latrine, water source and household assets, from EDHS 2016. Quintiles of the wealth index score was created to categorize households as Lower, Middle, and Higher.

**Functional status:** was categorized into three levels depending on the patient’s ability to carry out activities of daily living (fully independent, partially dependent, or totally dependent), according to the Katz Index of Independence in Activities of Daily Living [44].

### Data collection tools and procedures

Data were collected from adult surgical patients admitted to a surgical ward using a pretested, structured interviewer-administered questionnaire, medical record review, and direct measurement of BMI. The data collection instrument was adapted from previously published studies [8, 19, 20], whereas wealth index assessment questions adopted from EDHS 2016, and the functional status assessment tool was adopted from the Katz Index of Assessing Tool [44]. The questionnaire included sociodemographic information, wealth index assessment, admission-related information, clinical-related information, behavioral information, and the Katz Index of functional status assessment tools. The data collectors were four BSc nurse professionals and three MSc nurse supervisors. The collected data were checked daily for consistency and accuracy.

### Data quality control measures

To ensure consistency, the questionnaires were translated into Amharic and then back into English. In this study, two senior surgeons and two researchers assessed face validity. Data collectors and supervisors received one-day training from the principal investigator on data collection, study aim, questionnaire content, timely collection, ethical issues, and interview techniques. Continuous supervision was provided by the principal investigator and supervisors. The questionnaire was pre-tested on 5% (n = 23) of the sample at Debre Tabor Comprehensive Specialized Hospital (DTCSH) before actual data collection to check acceptability and consistency. Depending on the results of the pretest, necessary changes such as ambiguous words and unavailable laboratory tests were corrected to ensure clarity and completeness of the questionnaire. Finally, the data were cleaned, categorized, compiled and checked for completeness and accuracy before the start of EpiData version 4.6.0.

### Data processing and analysis

EpiData version 4.6.0 was used to code and enter data, which were exported to STATA version 14 (StataCorp, College Station, TX, USA) for analysis. Descriptive statistics such as frequency and percentage were used to summarize the study population. Continuous variables were expressed using measures of central tendency and variability such as the mean, the median and the interquartile range (IQR). A binary logistic regression model was fitted. A variable with a p-value < 0.25 was used to select potential candidates for multivariable analysis, and variables with a p-value < 0.05 were considered significantly associated with the outcome variable. An odds ratio (OR) with a 95% confidence interval (CI) was used as a measure of association. The results are presented in the tables and graphics. The presence of multicollinearity among the independent variables was checked using the variance inflation factor (VIF = mean 1.40). The Hosmer-Lemeshow test showed that the model fit the data well (p = 0.42).

## Results

### Sociodemographic characteristics of the participants

This study included 452 adult surgical patients, with a response rate of 99%. Approximately two-thirds, 63.05% (285), were male, and the median age was 38 years, with nearly a third, 29.42% (133), ranging in age from 25 to 34 years. The majority of these, 76.8% (347), were married and more than half, 57.1% (258), were rural residents. About half (47.35% (214)) of the participants were farmers, and one third of the study participants (33.85% (153)) were in the lower wealth index. Three-quarters, 75% (339), had health insurance “Table 2”.

**Table 2:** Sociodemographic characteristics of adult surgical patients admitted to surgical wards in Amhara Regional State Comprehensive Specialized Hospitals, Ethiopia, 2023 (n = 452).

Variables	Category	Frequency	Percent (%)
**Sex**	Male	285	63.05
	Female	167	36.95
**Residency**	Urban	194	42.9
	Rural	258	57.1
**Marital status**	Married	347	76.77
	Single	56	12.39
	Widowed	37	8.19
	Divorced	12	2.65
**Age (in years)** **(Median = 38)**	18–24	62	13.72
	25–34	133	29.42
	35–44	88	19.47
	45–54	68	15.04
	55–64	48	10.62
	≥65	53	11.73
**Occupational status**	Farmer	214	47.35
	Employed	80	17.7
	Merchant	49	10.84
	Housewife	34	7.52
	Student	40	8.85
	Daily labor	24	5.31
	Others ^a^	11	2.43
**Wealth index**	Lower	153	33.85
	Middle	151	33.41
	Higher	148	32.74
**Educational level**	No formal education	190	42.04
	Primary (1–8) school	99	21.9
	Secondary (9–12) school	67	14.82
	College and above	96	21.24
**Health insurance**	Yes	339	75
	No	113	25

^a^Retried, and jobless.

### Admission-related characteristics

Of the 452 study participants, almost two-thirds, 63.72% (288), had undergone elective surgery. The majorities, 82.74% (374), were admitted during the day and 72.35% (327) presented directly from home “Table 3”.

**Table 3:** Admission-related characteristics of adult surgical patients admitted to surgical wards in Amhara Regional State Comprehensive Specialized Hospitals, Ethiopia, 2023 (n = 452).

Variables	Category	Frequency	Percent (%)
**Mode of surgery admission**	Emergency	164	36.28
	Elective	288	63.72
**Time of admission**	Day time	374	82.74
	Night time	78	17.26
**Source of referral**	Home	327	72.35
	Public health facility	105	23.23
	Private health facility	20	4.42

### Clinical characteristics

Out of the 452 study participants, 4.65% (21) had postponed surgery and 11.06% (50) had comorbid conditions. During their hospital stay, 7.96% (36) developed hospital-acquired pneumonia (HAP). More than a third (38.94% (176)) of participants had surgeries lasting ≥110 minutes and 22.57% (102) had preoperative anemia. In addition, 17.92% (81) had low body mass index “Table 4”.

**Table 4:** Clinical features of adult admitted surgical patients in Amhara Regional State Comprehensive Specialized Hospitals, Ethiopia, 2023 (n = 452).

Variables	Category	Frequency	Percent (%)
**Postponed surgery**	Yes	21	4.64
	No	434	95.35
**Reason for postponed surgery (n = 21)**	Lack of blood for surgery	11	2.43
	Investigation not done	4	0.88
	Lack of funds	4	0.88
	Others ^a^	2	0.44
**Comorbidity**	Yes	50	11.06
	No	402	88.94
**Type of comorbid conditions (n = 57)**	Hypertension	18	3.98
	Diabetes mellitus	15	3.32
	Thyroid disease	13	2.87
	Others ^b^	11	2.43
**Type of Surgery procedures**	Upper and lower GIT	165	36.50
	Head	28	6.19
	Neck	86	19.03
	Urology	89	19.69
	Biliary tract	49	10.84
	Breast	14	3.10
	Others^c^	21	4.65
**Surgical site infection**	Yes	17	3.76
	No	435	96.24
**HAP**	Yes	36	7.96
	No	416	92.04
**Urinary tract infection**	Yes	10	2.21
	No	442	97.79
**Wound dehiscence**	Yes	14	3.10
	No	448	96.90
**Pressure ulcer**	Yes	7	1.55
	No	445	98.45
**Sepsis/septic shock**	Yes	7	1.55
	No	446	98.45
**DVT**	Yes	3	0.66
	No	449	99.34
**Reoperation**	Yes	23	5.09
	No	429	94.91
**Number of reoperation (n = 23)**	≤ 1	18	3.98
	>1	5	1.11
**Duration of surgery (minutes)**	≤70	161	35.62
	70–110	115	25.44
	≥110	176	38.94
**Preoperative anemia**	Yes	102	22.57
	No	350	77.43
**Body mass index**	Underweight	81	17.92
	Normal	336	74.34
	Overweight	35	7.74
**Functional status**	Independent	217	48.01
	Partially dependent	232	51.33
	Fully dependent	3	0.66

DVT, deep vein thrombosis; HAP, hospital acquired pneumonia.

^a^Holiday date, shortage of time.

^b^Peptic ulcer disease, stroke.

^c^Right leg psoas drainage, perianal drainage, fasciotomy, skin grafting and vascular surgery

### Behavioral characteristics of the study participants

Of the study participants, 41.59% (188) were currently consuming alcohol, whereas the majority, namely 90.71% (410) of the respondents stated that they had never smoked cigarettes “Table 5”.

**Table 5:** Behavioral characteristics of adult surgical patients admitted to surgical wards in Amhara Regional State Comprehensive Specialized Hospitals, Ethiopia, 2023 (n = 452).

Variables	Category	Frequency	Percent (%)
**Alcohol drinking status**	Never	133	29.2
	Current	188	41.59
	Past drinker	131	28.98
**Smoking status**	Never	410	90.71
	Current	29	6.42
	Former	13	2.88

### Length of hospital stay

**Fig 2:**
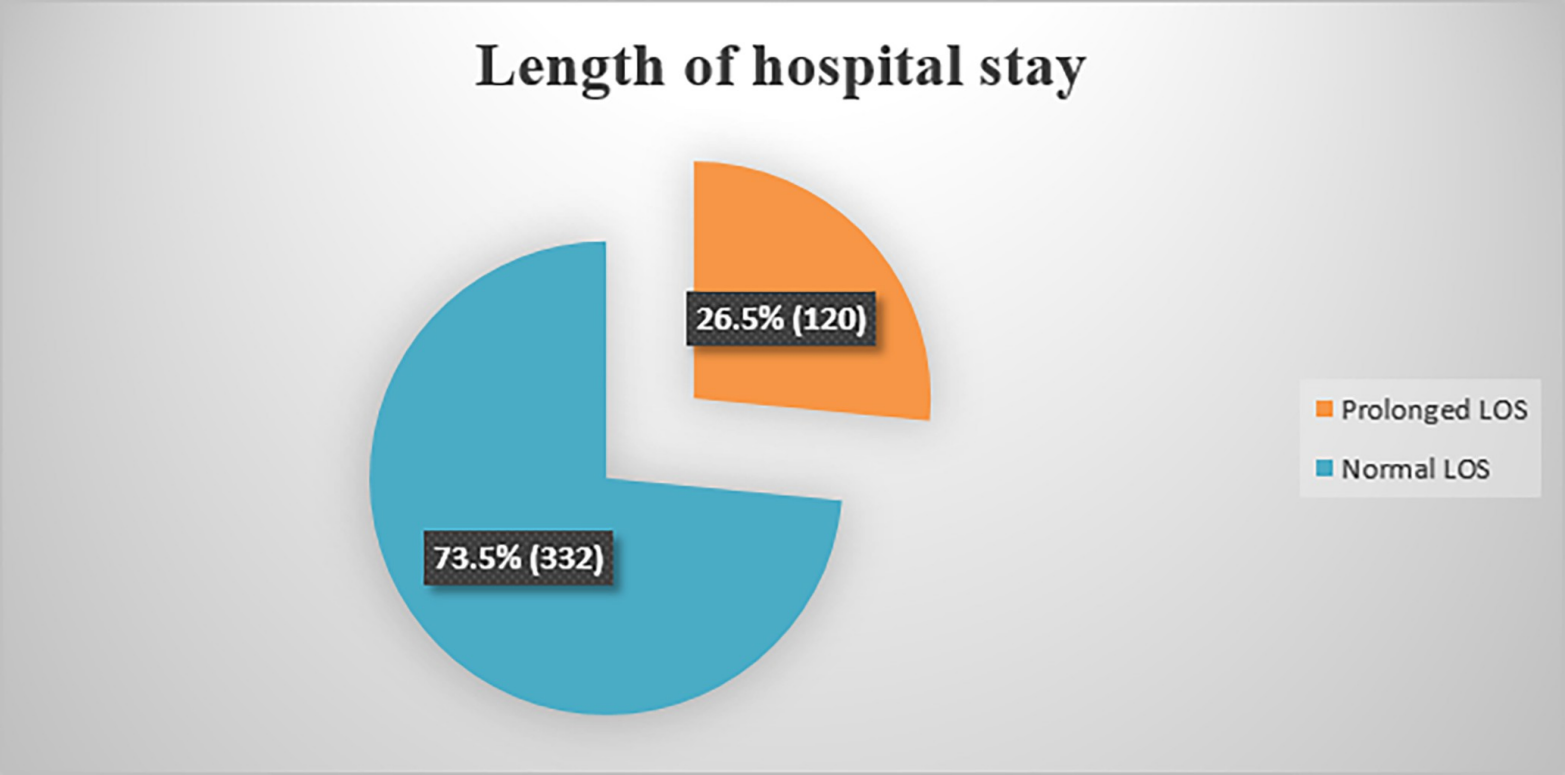
Proportion of length of hospital stays among adult surgical patients admitted to surgical wards in Amhara Regional State Comprehensive Specialized Hospitals, Ethiopia, 2023 (n = 452).

**Fig 3:**
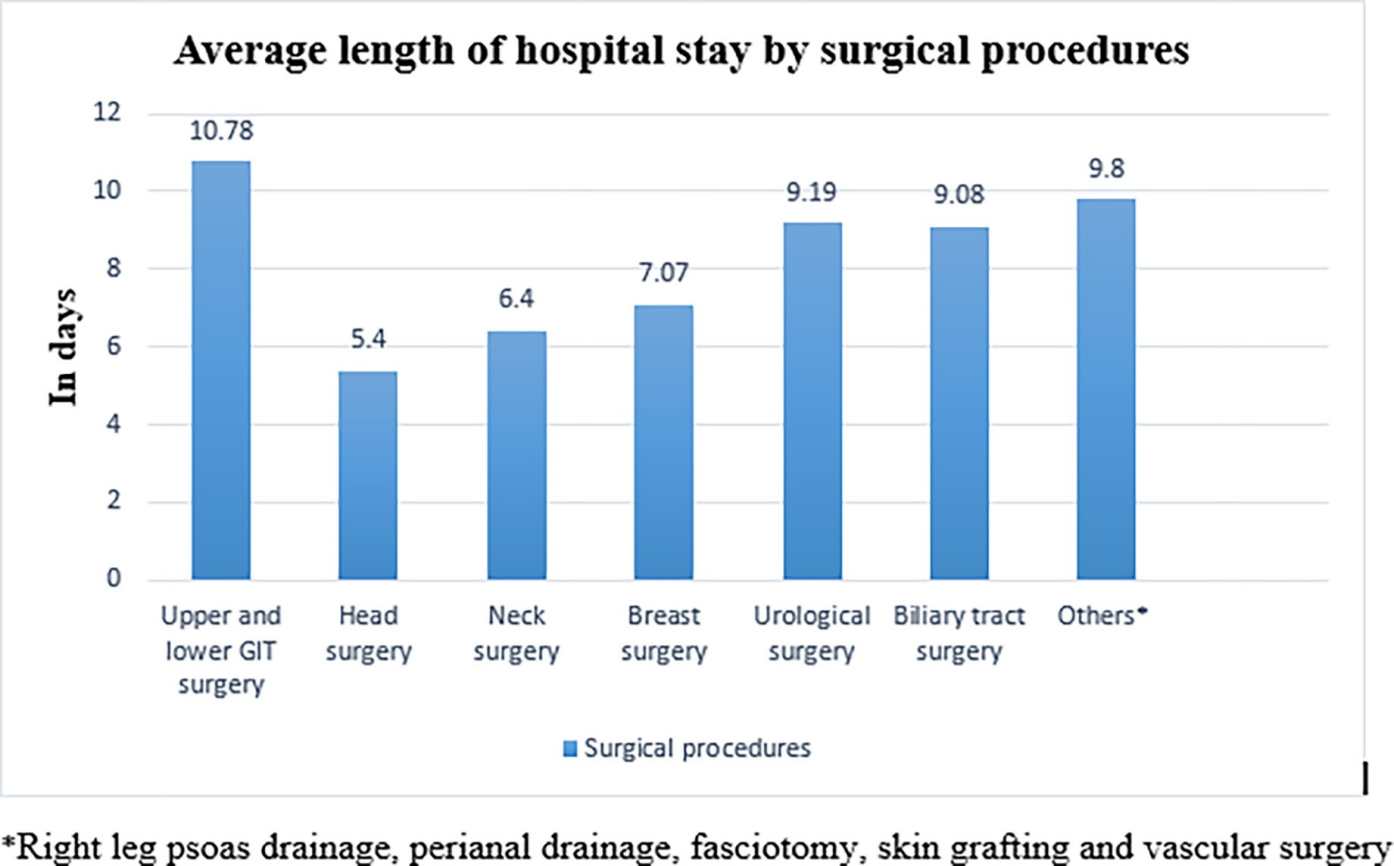
Average length of hospital stays among adult surgical patients admitted to surgical wards in Amhara Regional State Comprehensive Specialized Hospitals, Ethiopia, 2023 (n = 452).

In the current study, the prevalence of prolonged hospital stays was 26.5% (95% CI: 22.7–30.8). The average length of hospital stay was 9.38 (SD: ±7.31) days. The minimum and maximum duration of hospital stays were 2 and 60 days, respectively “Fig 2”. The highest average length of hospital stay was recorded in patients who underwent upper and lower gastrointestinal surgery, followed by others “Fig 3”.

### Factor associated with prolonged length of hospital stay

In the bivariate analysis, sex, marital status, health insurance, type of surgical admission, referral source, time of admission, body mass index, wealth index, HAP, surgical site infection (SSI), reoperation, preoperative anemia, alcohol status, and the duration of the Surgery were factors associated with prolonged LoS. However, in multivariable logistic regression analysis, only five variables (source of referral, body mass index, HAP, preoperative anemia, and duration of surgery) were significantly associated with prolonged LoS.

The odds of prolonged LoS were 2.65 times higher (AOR = 2.65; 95% CI: 1.14, 6.14) in patients referred from another public health facility than those directly were presented from home. In addition, the odds of prolonged LoS were 3.6 times higher (AOR = 3.64; 95% CI: 1.43, 9.23) in patients who developed hospital-acquired pneumonia than in patients who had no HAP. Moreover, the odds of prolonged LoS were 2.5 times higher (AOR = 2.54; 95% CI: 1.25, 5.16) in patients whose surgery lasted ≥ 110 minutes than in patients whose surgery duration ≤ 70 minutes. Additionally, the odds of prolonged LoS were 5.2 times higher (AOR = 5.21; 95% CI: 2.63, 10.33) in underweight patients than in normal weight patients. Furthermore, the odds of prolonged LoS were 3.2 times higher (AOR = 3.22; 95% CI: 1.77, 5.86) in patients with preoperative anemia than in patients without preoperative anemia “Table 6”.

**Table 6:** Bivariable and multivariable analysis of factors associated with the length of hospital stay among adult surgical patients admitted to surgical wards in Amhara Regional State Comprehensive Specialized Hospital, Ethiopia, 2023 (n = 452).

Variables	Length of hospital stays		COR (95% CI)	AOR (95% CI)
	Prolonged No. (%)	Normal No. (%)		
**Sex**				
Male	91 (31.93)	194 (68.07)	2.23 (1.39, 3.57)	1.73 (0.93, 3.21)
Female	29 (17.37)	138 (82.63)	1	1
**Marital status**				
Married	81 (23.4)	266 (76.66)	1	1
Single	23 (41.07)	33 (58.93)	2.28 (1.27, 4.12)	1.71 (0.75, 3.89)
Widowed	11 (29.73)	26 (70.27)	1.38 (0.65, 2.93)	1.83 (0.66, 5.06)
Divorced	5 (41.67)	7 (58.33)	2.34 (0.72, 7.59)	3.10 (0.62, 10.28)
**Wealth index**				
Lower	55 (35.95)	98 (64.05)	2.76 (1.61, 4.74)	1.33 (0.54, 3.23)
Middle	40 (26.49)	111 (73.51)	1.77 (1.01, 3.10)	1.03 (0.45, 2.36)
Higher	25 (16.89)	123 (83.11)	1	1
**Health insurance**				
No	25 (22.12)	88 (77.88)	1	1
Yes	95 (28.02)	244 (71.98)	1.37 (0.82, 2.27)	1.16 (0.51, 2.65)
**Mode of surgery**				
Elective	51 (17.71)	237 (82.29)	1	1
Emergency	69 (42.07)	95 (57.93)	3.37 (2.19, 5.21)	0.86 (0.34, 2.11)
**Time of admission**				
Day time	80 (21.39)	294 (78.61)	1	1
Night time	40 (51.28)	38 (48.72)	3.87 (2.32, 6.43)	1.18 (0.53, 2.64)
**Source of referral**				
Home	59 (18.04)	268 (81.96)	1	1
Public health facility	54 (51.43)	51 (48.57)	4.80 (2.99, 7.73)	**2.65 (1.15, 6.14)** *
Private health facility	7 (35)	13 (65)	2.44 (0.93, 6.39)	1.88 (0.47, 7.51)
**Body mass index**				
Normal	74 (22.02)	262 (77.98)	1	1
Underweight	34 (41.98)	47 (58.02)	2.56 (1.53, 4.27)	**5.21 (2.63, 10.33)** **
Overweight	12 (34.29)	23 (65.71)	1.84 (0.87, 3.88)	2.24 (0.88, 5.68)
**HAP**				
No	98 (23.56)	318 (76.44)	1	1
Yes	22 (61.11)	14 (38.89)	5.09(2.51, 10.34)	**3.64 (1.43, 9.23)** *
**Surgical site infection**				
No	110 (25.29)	325 (74.71)	1	1
Yes	10 (58.82)	7 (41.18)	4.22(1.56, 10.22)	1.49 (0.38, 5.83)
**Duration of surgery(minutes)**				
≤70	25 (15.53)	136 (84.47)	1	1
70–110	15 (13.04)	100 (86.96)	0.816(0.41, 1.63)	0.87 (0.32, 1.64)
≥110	80 (45.45)	96 (54.55)	4.53 (2.69, 7.62)	**2.54 (1.25, 5.16)** *
**Reoperation**				
No	108 (25.17)	321 (74.83)	1	1
Yes	12 (52.17)	11 (47.83)	3.24 (1.39, 7.56)	0.63 (0.21, 1.93)
**Preoperative anemia**				
No	67 (19.14)	283 (80.86)	1	1
Yes	53 (51.96)	49 (48.04)	4.57 (2.85, 7.31)	**3.22 (1.77, 5.86)** **
**Alcohol status**				
Never	28 (21.05)	105 (78.95)	1	1
Current	75 (39.89)	113 (60.11)	2.48 (1.49, 4.14)	1.21 (0.61, 2.41)
Past drinker	17 (12.98)	114 (87.02)	0.55 (0.28, 1.08)	0.54 (0.24, 1.27)

HAP, hospital acquired pneumonia; CI, confidence interval; AOR, adjusted odds ratio; COR, crudes odds ratio.

*P value < 0.05

**P value < 0.001; and 1-reference.

## Discussion

Assessing the length of hospital stay of surgical patients admitted to the surgical ward is one of the most useful approaches to assess the quality of surgical treatment of patients. Furthermore, identifying patients with longer hospital stays could be a useful strategy to reduce unnecessary length of stay (LoS) in hospitals.

According to the findings of the current study, 26.5% of the patients experienced prolonged LoS. This finding is consistent with previous studies conducted in Oman (30.5) [9], Boston (27.7%) [45], and Jimma University Medical Center (JUMC) (25.3%) [19]. However, higher than the study conducted in Australia (9.7%) [14], Philippines (19.17%) [18], the USA (22%) [46], 18 Polish and German surgical centers (4.92%) [47], and Saudi Arabia (21%) [48]. This discrepancy may be due to advances in the healthcare system and the fact that specialized healthcare professionals provide the majority of care in developed countries, which is difficult to implement in low- and middle-income countries. Additionally, studies have shown that developing countries face numerous barriers to providing quality surgical care [49].

On the other hand, this study result is lower than that of the studies conducted in Nigeria (63%) [20] and Thailand (54.9%) [50]. In the case of Nigeria, this discrepancy may be due to the study period and the lower cutoff values, which were at least seven days, which is less than the cutoff used in this study. In Thailand, the difference could be due to the source population; the study was conducted on patients with a fractured femoral neck. According to a study, patients with fractures had a longer hospital stay than other surgical patients [51].

The current study found that source of referral, preoperative anemia, body mass index, HAP, and duration of surgery were significantly associated with prolonged LoS. This study found that patients referred from another public health facility were more likely to have a prolonged hospital stay than those who came directly from home. This finding is supported by previous studies conducted in Australia and the USA [28, 52]. There was no clear reason why the referred patients experienced prolonged LoS. However, a possible explanation could be that due to the complexity of treatment, referred patients may require more time for investigations and treatment, as they may be sicker than non-referred patients. These factors led to an increase in the duration of treatment and examination, which in turn led to an increase in their hospital stay. An important point emerging from the current results is that the source of referral is one of the factors leading to longer hospital stays.

This study found that underweight patients were more likely to have prolonged LoS than normal weight patients. A similar study from Ethiopia supports this finding [53]. This finding may be explained by the fact that patients with inadequate nutritional status had an impact on the typical wound healing stage. This leads to long-term treatment and prolonged LoS. This result holds implications for underweight increasing the LoS of patients.

The current study found that patients who had preoperative anemia were 3.2 times more likely to have a prolonged hospital stay than those without preoperative anemia. Similar studies from Singapore and Germany support this study [30, 40]. This may be due to untreated preoperative anemia related to complications following a surgical procedure as well as a higher demand for blood transfusion and hence prolonged LoS [30]. An additional explanation may be that patients with anemia frequently experience fatigue and dizziness, which can make it difficult for them to participate in their own therapy. As a result, this may extend the hospital stay. Studies have shown that early detection of anemia is essential in the context of surgical planning (at least 2–4 weeks prior to surgery). In emergency cases, parenteral substitution with iron directed at the end of surgery or postoperatively, especially in cases of perioperative blood loss of more than 500 ml, can improve treatment outcomes [40]. This finding suggests that preoperative anemia tends to extend the hospital stay for patients.

This study revealed that patients with hospital-acquired pneumonia were 3.6 times more likely to have prolonged LoS than those without HAP. This finding is consistent with that of a study conducted in the USA [45]. This may be related to the fact that the innate immune system predominantly directs its cells to fight lung infections and shows a delayed response to wound healing, which in turn leads to longer treatment duration and longer hospital stay [54]. The findings have implications for the presence of hospital-acquired pneumonia and how this affects patients’ length of hospital stay.

A recent study found that patients with an operation time of ≥110 minutes were 2.5 times more likely to have a prolonged LoS than those with an operation time of ≤70 minutes. This result is supported by several previous studies conducted in Japan, China and the USA [22, 31, 55]. The possible reason could be that longer operation time is usually related to more complicated case, higher risk of intraoperative complications and increased estimated blood loss (EBL). These factors, in turn, can affect the patient’s recovery and lead to a longer hospital stay [22]. This finding suggests that prolonged surgery is a significant factor prolonging the length of hospital stay.

### Strength and limitation of the study

The strength of this study is that it is the first in this research area to provide information about LoS and associated factors in surgical patients admitted to surgical wards. Furthermore, it was a multicenter study with a large sample size. The limitations of this study were social desirability and recall bias. These were minimized through a detailed explanation of the study objectives, anonymous use, separately conducted interviews and chart reviews.

## Conclusion

This study found that a significant proportion of prolonged hospital stays occur among patients on adult surgical wards. Patients referred from another public health facility, preoperative anemia, underweight, duration of surgery ≥110 minutes, and hospital-acquired pneumonia were factors associated with prolonged hospital stay. Before surgery, screening and treatment of patients with anemia and malnutrition is advisable. Healthcare providers should ensure that referred patients have received the necessary treatments and investigations in hospitals. Additionally, standard safety checklists and infection prevention precautions should be used to improve the quality of care. Future researchers should consider additional hospital-related controllable factors and use mixed methods to identify additional factors associated with prolonged hospital stays.

## Abbreviations

AOR: adjusted odds ratio
BMI: body mass index
CI: confidence interval
COR: crudes odds ratio
HAP: hospital acquired pneumonia
LoS: length of stay
PLoS: prolonged length of stay
USA: United States of America
